# Tomato Yellow Leaf Curl Sardinia Virus Increases Drought Tolerance of Tomato

**DOI:** 10.3390/ijms24032893

**Published:** 2023-02-02

**Authors:** Camilla Sacco Botto, Slavica Matić, Amedeo Moine, Walter Chitarra, Luca Nerva, Chiara D’Errico, Chiara Pagliarani, Emanuela Noris

**Affiliations:** 1 Institute for Sustainable Plant Protection, National Research Council, Strada delle Cacce 73, 10135 Turin, Italy; 2 Department of Agriculture, Forestry and Food Science DISAFA, Turin University, Largo Braccini 2, 10095 Grugliasco, Italy; 3 Council for Agricultural Research and Economics Centre of Viticultural and Enology Research (CREA-VE), Viale XXVIII Aprile 26, 31015 Conegliano, Italy

**Keywords:** *Solanum lycopersicum*, geminivirus, hormone signaling, recovery, gene expression

## Abstract

Drought stress is one of the major physiological stress factors that adversely affect agricultural production, altering critical features of plant growth and metabolism. Plants can be subjected simultaneously to abiotic and biotic stresses, such as drought and viral infections. Rewarding effects provided by viruses on the ability of host plants to endure abiotic stresses have been reported. Recently, begomoviruses causing the tomato yellow leaf curl disease in tomatoes were shown to increase heat and drought tolerance. However, biological bases underlying the induced drought tolerance need further elucidation, particularly in the case of tomato plants. In this work, tomato plants infected by the tomato yellow leaf curl Sardinia virus (TYLCSV) were subjected to severe drought stress, followed by recovery. Morphological traits, water potential, and hormone contents were measured in leaves together with molecular analysis of stress-responsive and hormone metabolism-related genes. Wilting symptoms appeared three days later in TYLCSV-infected plants compared to healthy controls and post-rehydration recovery was faster (2 vs. 4 days, respectively). Our study contributes new insights into the impact of viruses on the plant’s adaptability to environmental stresses. On a broader perspective, such information could have important practical implications for managing the effects of climate change on agroecosystems.

## 1. Introduction

Fast changing climate conditions have a significant influence on agricultural lands, raising CO_2_ and temperature levels while reducing water availability, with severe consequences for plant development and crop yields [1]. A shortage of water availability is one of the most severe abiotic stresses to which plants are forced to respond in order to restore cellular equilibrium and enhance their survival. Plant responses to drought stress aim at regulating the stomatal opening to limit water loss through transpiration. Hormonal balances and antioxidant activities constitute important hubs in the adaptation of plants to water stresses [2].

Recent studies related to plant—virus interactions have revealed that, beside known detrimental effects, these pathogens might increase the plant’s ability to withstand abiotic stresses, altering host metabolism and development [3,4,5]. In an evolutionary perspective of plant—pathogen interaction, this cross-talk phenomenon [6] indicated the occurrence of cross-regulative responses towards stresses via synergistic or antagonistic actions. The first records of viral mutualistic symbiosis reported that a few viruses induced abiotic stress tolerance in the model host *Nicotiana benthamiana* and in different crops [4,5,7,8]. Since then, other examples of positive impact of plant viruses towards their hosts have been published, reviewed in [9,10], opening up new concepts and strategies to improve plant tolerance to severe temperature increase and drastic water decline, relevant in the context of the ongoing climate change. One of the best-studied cases of such cross-talk is represented by simultaneous viral infection and drought stress, a combination where a wide range of signaling components, such as hormones, proteins, transcription factors, and/or membrane and cell wall receptors eventually lead to the expression of different but partially overlapping sets of genes [11].

The tomato (*Solanum lycopersicum* L.) is the most important vegetable crop in Italy, a country that ranks among the six major producers in the world [12]. Tomatoes can be attacked by several viruses, including members of the genus *Begomovirus* (family *Geminiviridae*) which are spread in the Mediterranean regions and in several subtropical areas causing tomato yellow leaf curl disease (TYLCD) [13]. A few studies have recently investigated the role of tomato yellow leaf curl virus (TYLCV), one of the begomoviruses inducing TYLCD, to promote heat and drought tolerance in plants, in spite of the severe disease caused under normal irrigation conditions. In particular, TYLCV infection was shown to support an increased heat tolerance in laboratory- and field-grown tomato plants [14]. Moreover, it was reported that TYLCV-infected plants had increased drought stress tolerance [15] and survived longer than uninfected ones under water deficit conditions, possibly thanks to a more developed root system [16,17,18]. However, those studies mainly focused on virus-related changes occurring in the regulation of cell homeostasis in plants subjected to heat and drought stress, while issues associated with hormone signaling and metabolism or with anatomical traits still remain unanswered.

To more deeply investigate if the association of tomato with begomoviruses improves drought stress tolerance, here we focused on the tomato yellow leaf curl Sardinia virus (TYLCSV), another TYLCD-inducingvirus, widespread in the western part of the Mediterranean basin. TYLCSV has a monopartite single-stranded DNA genome of 2773 nucleotides, harboring six partially or entirely overlapping genes which encode the coat protein (CP), the V2 protein, the replication-associated protein (Rep-C1), and the C2, C3, and C4 proteins. The role of the C4 protein has been linked to pathogenicity, viral movement, and silencing suppression [19]. Recently, we reported that the overexpression of the TYLCSV-C4 gene in transgenic tomato plants led to an increased ability to tolerate drought, delaying the onset of stress features, ameliorating plant water use efficiency, and promoting faster recovery dynamics; in the same study, these physiological responses were found to occur in association with specific modifications at the anatomic and metabolic levels [20].

Therefore, to study if TYLCSV modulates *per se* the endurance of tomato plants exposed to complete water deprivation, uninfected and TYLCSV-infected plants subjected to a drought stress and recovery time-course were compared, analyzing transcriptional changes associated with water stress defence pathways, together with alterations in the content of key phytohormones involved in the stress response. In addition, specific anatomical traits were investigated. Collectively, substantial evidence regarding the beneficial effect of TYLCSV infection not only in mitigating the negative impact of drought but also in accelerating the recovery timing after rehydration of tomato plants was provided, and new aspects of the hormone-based cross-talk between abiotic stress and virus presence were investigated.

## 2. Results and Discussion

### 2.1. Drought Stress Perception Is Attenuated in TYLCSV-Infected Plants

**Figure 1:**
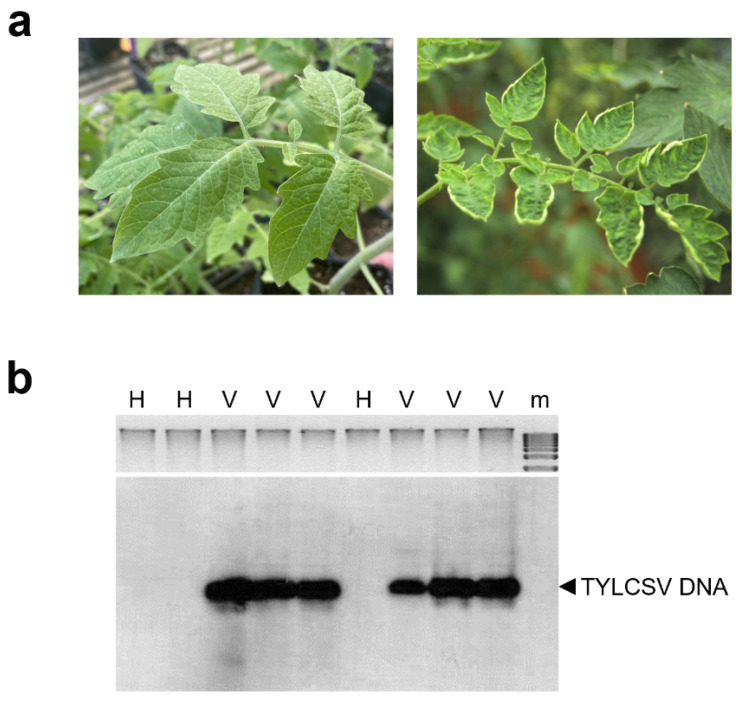
Determination of viral symptoms and of viral genome presence in tomato (cv. Moneymaker) plants. (**a**) Symptoms on leaves of a mock inoculated (**left**) or tomato yellow leaf curl Sardinia virus (TYLCSV)-infected (**right**) plants, photographed 2 months after inoculation (mpi). (**b**) Southern blot hybridization of mock (H) or TYLCSV (V) inoculated plants, analyzed at 2 mpi, before starting the drought stress trial. A coat protein-gene specific probe was used for virus detection. The arrow indicates the genomic single-stranded TYLCSV DNA. Ethidium bromide staining of plant genomic DNA (corresponding to 200 ng per lane) is shown as loading control. m = 1 kb ladder.

In order to characterize the impact of TYLCSV infection on the response of tomato plants to water deprivation, a group of twelve plants infected by TYLCSV were used. These plants, obtained by inoculating a TYLCSV agroinfectious clone, exhibited typical leaf yellowing and curling symptoms, starting from 4 weeks after inoculation (wpi) (Figure 1a), in agreement with previous observations [21,22]. Moreover, before starting the drought stress trial, these plants tested positive with Southern hybridization analysis performed with a TYLCSV-specific probe (Figure 1b). In parallel, a group of twelve uninfected control plants (mock) were prepared, showing neither virus symptoms (Figure 1a) nor positive hybridization signals (Figure 1b).

**Figure 2:**
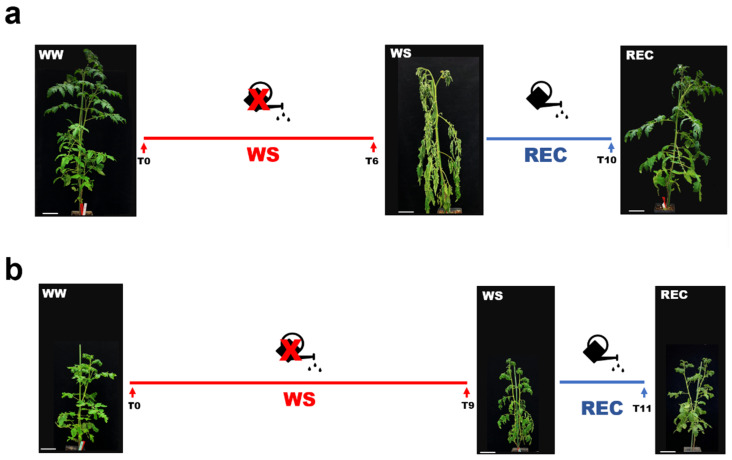
Impact of TYLCSV infection on drought stress perception of tomato plants. Schematic representation of the drought stress and recovery time-course in (**a**) mock-inoculated and (**b**) TYLCSV-infected plants. The T letter followed by the number (e.g., T0) refers to as a specific day of the experimental trial. For each timeline, representative images of plants under irrigation (WW), at severe drought stress conditions (WS, i.e., at 6 and 10 days after water withdrawal for mock-inoculated and TYLCSV-infected plants, respectively), and at complete recovery (REC, i.e., at 4 and 2 days after rewatering for mock-inoculated and TYLCSV-infected plants, respectively) are shown. The bar at the bottom in each image corresponds to 10 cm.

To monitor the impact of TYLCSV on drought stress resilience, six virus-infected and six mock-inoculated plants were subjected to complete water withdrawal, while two corresponding groups of six plants each received daily watering. Plants were monitored daily for drought symptoms and, when clear wilting and plant collapse were reached, they were rewatered and allowed to recover (Figure 2).

Uninfected plants started to show wilting symptoms three days after water withdrawal and, by six days after starting the drought stress experiment, all plants were dramatically collapsed (Figure 2a). Conversely, TYLCSV-infected plants displayed wilting conditions nine days after the beginning of the treatment, but none of them showed the same drastic symptoms of the water-stressed (WS) mock plants (Figure 2b). Moreover, after rewatering, uninfected plants restored to a normal phenotype in 4 days, while TYLCSV-infected plants recovered to the initial pre-stress conditions in only 2 days (Figure 2a,b).

**Figure 3:**
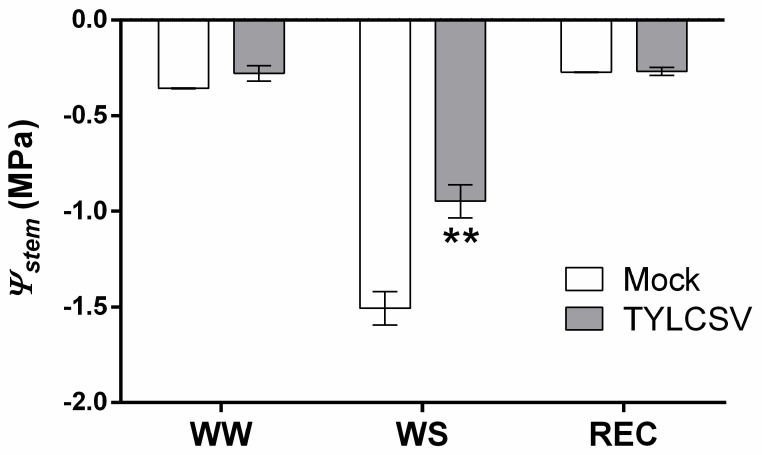
Measurement of xylem pressure values. Stem xylem pressure (Stem water potential, *Ψ_stem_*) of mock-inoculated and TYLCSV-infected plants was measured at the different water regimens (WW = Day 1; WS = Day 6 and Day 9; and REC = Day 10 and Day 11 of the time-course reported in Figure 2). The asterisks denote significant differences between the two sanitary conditions in the same watering status, as determined by a two-tailed Student’s *t* test (** *p* ≤ 0.01). Data are the mean ± SE (*n* = 6).

To evaluate the level of stress perception of the plants used in this experiment, stem water potential was measured on (i) daily irrigated plants (well-watered, WW), (ii) WS plants when visible drought stress conditions and severe wilting appeared, and (iii) after visible recovery (REC). As shown in Figure 3, in WW conditions, infected and uninfected plants had similar levels of stem water potential (*Ψ_stem_* −0.28 vs. −0.36 MPa, respectively). Following WS imposition, mock-inoculated plants reached a water potential of −1.51 MPa, a value significantly reduced compared to TYLCSV-infected individuals (−0.95 MPa). After rewatering, both groups of plants recovered to initial *Ψ_stem_* levels, without showing statistically significant differences (REC plants, Figure 3).

Overall, these data indicate that the TYLCSV infection induces a lower drought stress perception in the tomato, confirming previous observations made on *Arabidopsis*, *Nicotiana benthamiana* and the tomato with the related begomovirus TYLCV [15,16,17]. In addition, we also documented that TYLCSV-infected tomato plants exhibit a faster recovery after WS imposition, delaying plant entry into a severe stress condition by up to three days, a result with relevant agronomic implications.

### 2.2. TYLCSV Infection Alters the Morphology of Tomato Plants, Affecting Their Response to Water Stress

**Figure 4:**
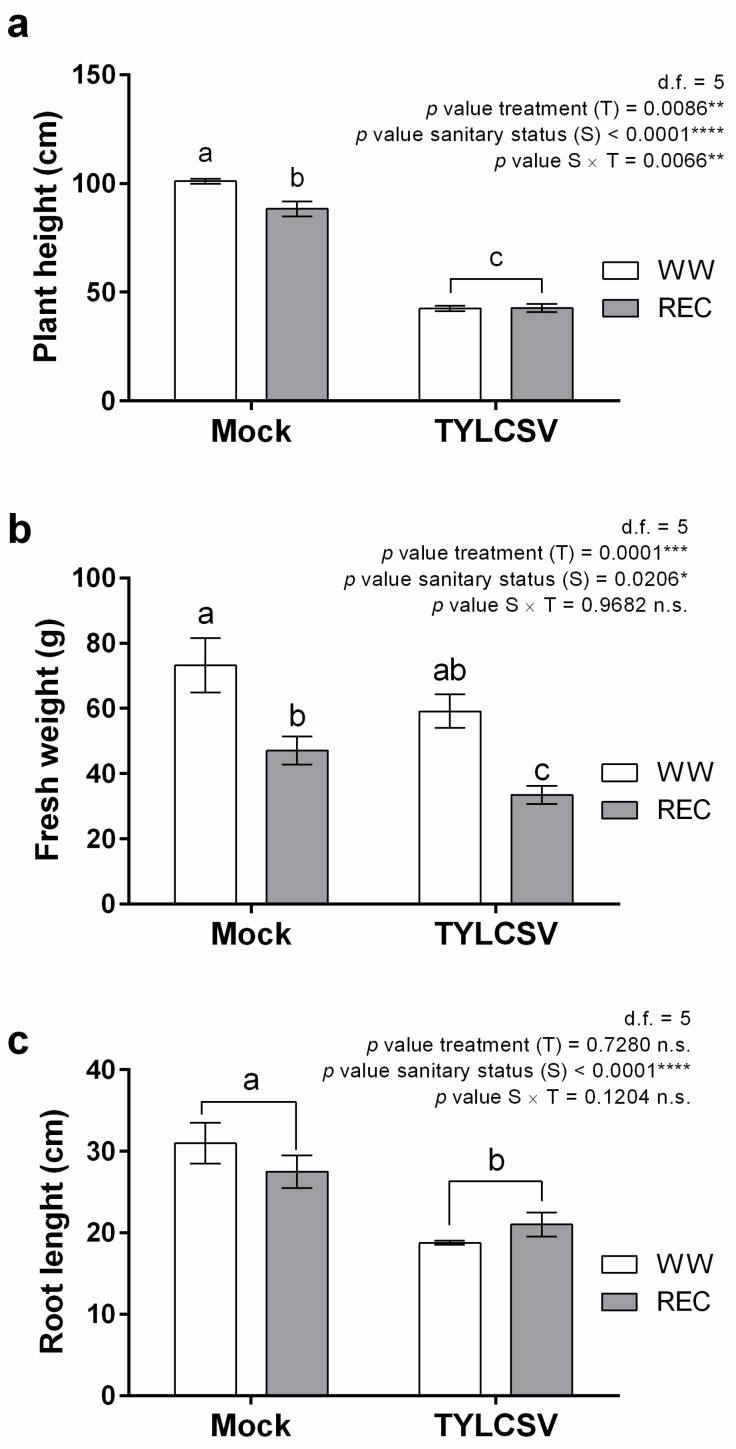
Biometric data analysis. Combined impact of tomato yellow leaf curl Sardinia virus (TYLCSV) infection and drought stress on (**a**) plant height, (**b**) fresh shoot weight, and (**c**) root length of well-watered plants (WW) and of plants subjected to drought stress and rewatering (REC). The significance of treatment (T), sanitary status (S), and sanitary status x treatment (S × T) interaction was assessed by Tukey’s *HSD* test and the corresponding results are given above each graph in the figure panel; *p* ≤ 0.05 (*); *p* ≤ 0.01 (**); *p* ≤ 0.001 (***); *p* ≤ 0.0001 (****); n.s. = not significant. Lower case letters are reported when the S × T interaction and/or sanitary status (S) main effects are statistically significant, as attested by Tukey’s *HSD* or Student’s *t* test, respectively. Data are the mean ± SE (*n =* 6).

Considering morphological parameters potentially responsible for the improved drought stress performance of TYLCSV-infected plants, plant height, fresh shoot weight, root length, and number of leaves were measured, comparing data taken from normally watered plants at the beginning of the experiment and from recovered plants at the end of the trial, in both sanitary conditions. TYLCSV-infection induced a 58% reduction in plant height in WW conditions (Figure 4a), without affecting the number of leaves (not shown). Interestingly, REC uninfected plants exhibited a significant decrease (13%) in plant height compared to unstressed plants, while no such effect occurred for infected individuals (Figure 4a).

In agreement with these results, TYLCSV-infected plants showed a significant decrease in fresh shoot weight compared to uninfected individuals in WW conditions (Figure 4b). Following WS, fresh shoot weight of REC plants decreased in both uninfected and infected plants (Figure 4b), but the effect of the treatment was more pronounced in uninfected compared to infected plants (64 vs. 56%, respectively) (Figure 4b).

Since the root apparatus is fundamental for the plant water management and since geminiviruses can influence its development [17], the root length of plants recovered after water deprivation (REC) was measured. Under normal irrigation, the root length of infected plants was significantly reduced (39%) compared to mock-inoculated controls, in agreement with the reduced plant size (Figure 4c). Interestingly, while the root length of REC uninfected plants showed a slight but non-significant decrease, no such effect occurred for REC infected plants compared to WW controls (Figure 4c), suggesting that infection partially protected plants from WS damage. Indeed, the only statistically significant effect was ascribed to the sanitary status of the plants (Figure 4c).

These results are in line with the recent observations made on TYLCV-infected tomato plants, having a 20–25% significant reduction of root dry weight and an increased root weight in the case of infected water stressed plants [17].

**Figure 5:**
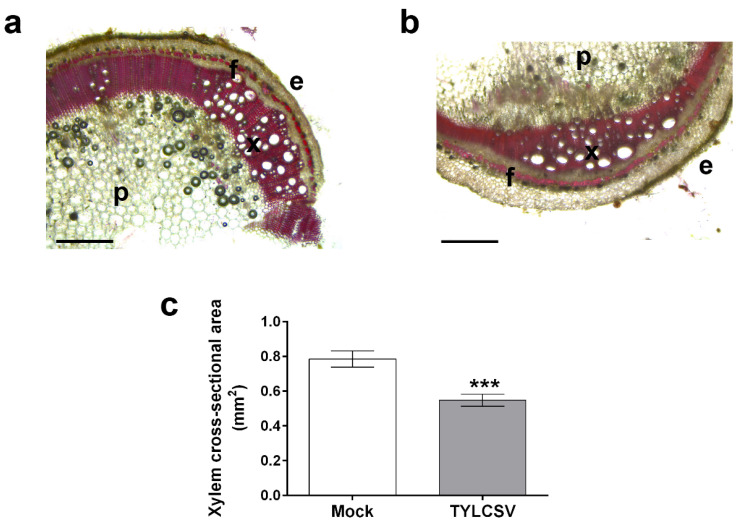
Analysis of the stem xylem area. Stem cross-sectional area of well-watered (**a**) uninfected (mock) and (**b**) TYLCSV-infected plants following safranin staining; e: epidermis, f: fibers, p: parenchyma, and x: xylem. (**c**) Whole xylem cross-sectional area of stems from mock-inoculated and TYLCSV-infected. The asterisks denote significant differences as attested by a tow-tailed Student’s *t* test (*** *p <* 0.001). Bars represent SE (*n* = 12). Magnification bars correspond to 200 µm.

Beside the invasion of plant viruses in the phloem tissue, several studies have demonstrated their presence in the xylem vasculature [23], with possible alteration of the xylem patterning mediated by vascular associated pathogens [24]. Notably, we recently reported that tomato plants overexpressing the TYLCSV-C4 protein show specific modifications at the xylem level, possibly contributing to their increased drought tolerance [20]. Moreover, overexpression of the C4 protein of the related TYLCV was reported to perturb xylem patterning through its interaction with receptor-like kinases in Arabidopsis [25]. Therefore, to evaluate if TYLCSV infection perturbs the xylem in a way similar to the effect produced by the expression of its C4 protein, transversal stem sections of virus-infected plants were analyzed under optical microscope. As displayed in Figure 5, compared to uninfected plants (Figure 5a), the entire xylem cross-sectional area was significantly narrower in infected plants (Figure 5b), attesting a significant reduction of 30% (Figure 5c).

It is well documented that structural changes affecting xylem vessel size and organization can contribute to regulating its hydraulic efficiency [26], also influencing the plant’s vulnerability to drought stress [27]. Specifically, it was reported that a reduction in stem xylem vessel transectional area could prevent serious drought effects, such as the risk of embolism formation, thereby facilitating water transport upon severe water stress conditions [28]. Unlike mock-inoculated plants, the anatomical adjustments observed at the xylem level in TYLCSV-infected plants (Figure 5) could most likely help them to endure drought stress pressure by limiting excessive water loss, in turn supporting their delayed response to stress occurrence (Figure 2b).

### 2.3. Hormonal Content Is Altered in TYLCSV-Infected Plants

**Figure 6:**
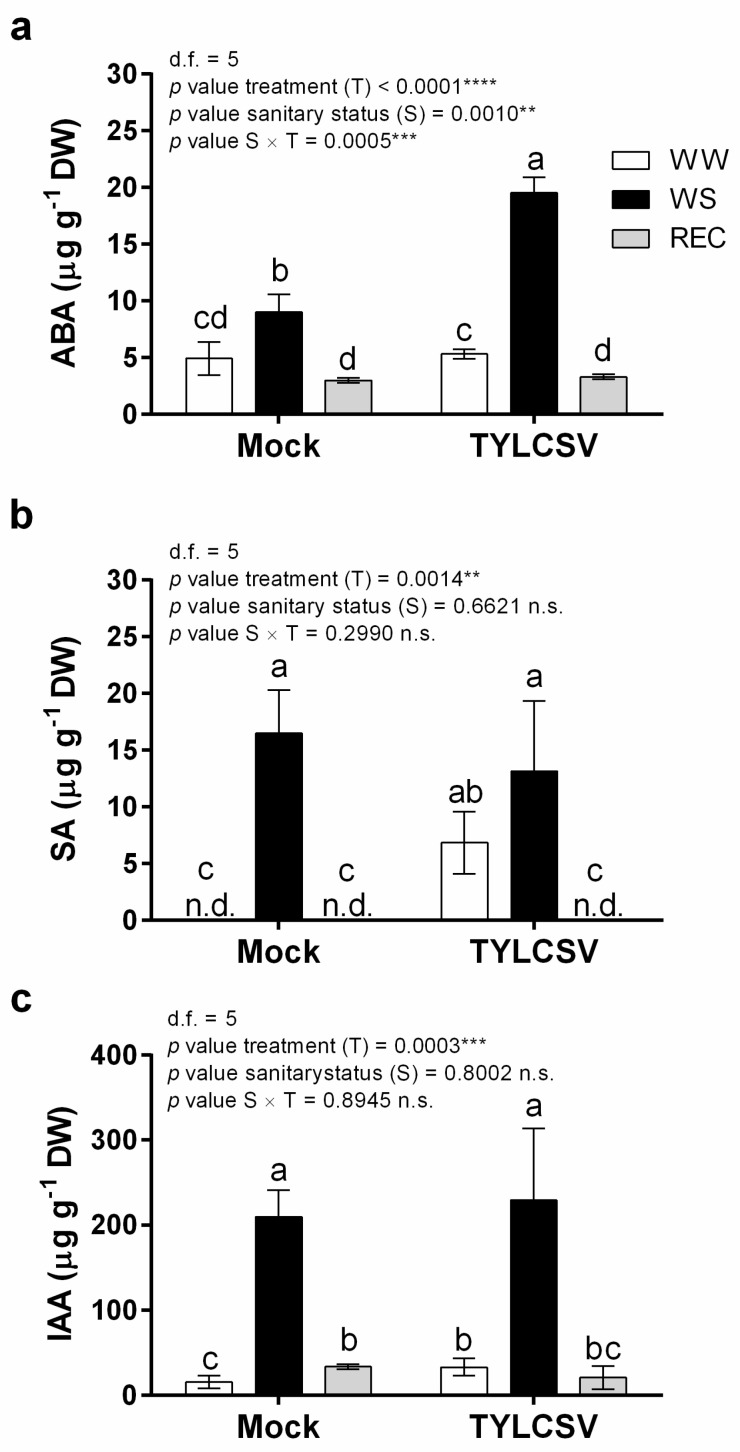
Content of stress-associated phytohormones. Differential impact of TYLCSV-infection and drought stress imposition on hormone concentrations. Content of (**a**) abscisic acid (ABA), (**b**) salicylic acid (SA), and (**c**) indole 3-acetic acid (IAA) in leaf samples of uninfected (mock) and TYLCSV-infected tomato plants under well-watered conditions (WW) or subjected to water stress treatment (WS), followed by re-watering (REC). The significance of treatment (T), sanitary status (S), and sanitary status × treatment (S × T) interaction was assessed by Tukey’s *HSD* test and the corresponding results are given above each graph in the figure panel; *p* ≤ 0.01 (**); *p* ≤ 0.001 (***); *p* ≤ 0.0001 (****); n.s. = not significant. Lower case letters are reported when the S × T interaction and/or sanitary status (S) main effects are statistically significant, as attested by Tukey’s *HSD* or Student’s *t* test, respectively. Data are the mean ± SE (*n* = 3).

Phytohormones are important endogenous chemical messengers modulating not only growth and development of plants, but also their response to negative stress conditions. Considering the dual role of hormones during geminivirus infection [29] and drought stress perception [30,31], we next investigated the hormonal regulation in uninfected and infected plants exposed to complete water deprivation, followed by rewatering (Figure 6).

In plants, ABA is the most important phytohormone mediating the responses to abiotic stresses, such as drought and temperature [32]. Its fundamental role in the response to water scarcity primarily relies on stomata closure to limit water loss through transpiration and allow a better water transport [30]. In our experiments, ABA accumulation in leaves followed a similar trend in both uninfected and infected plants, being strongly increased during water stress and restored to initial levels after rewatering (Figure 6a). However, while the ABA levels were similar in mock-inoculated and TYLCSV-infected plants in normal watering conditions, WS infected plants accumulated significantly more ABA (corresponding to a 54% increase) than uninfected controls (Figure 6a). Such an increase could contribute to limiting excessive water loss, supporting the higher tolerance of infected plants to water deprivation (Figure 2), in line with their delayed response to drought treatment and their higher *Ψ_stem_* values (Figure 3). Additionally, whether these findings are considered with those recently published on TYLCSV C4-overexpressing (OE) plants [20], it emerged that despite similar morphometric and anatomical adjustments (e.g., reduced xylem vessel area in both C4-OE and TYLCSV-infected plants), a different regulation of biochemical signals was established in TYLCSV-infected plants following WS. In fact, compared to controls, ABA accumulation patterns strongly increased upon drought in the infected condition (this work), while ABA significantly decreased in C4-OE tomatoes exposed to the same treatment [20]. These results thus suggest that still to be defined endogenous signaling pathways, either linked to the virus presence or the viral transgene OE, could intervene to modulate the plant physiological response to stress.

SA has an important role during stress and antiviral defense, intervening both at the local level in the hypersensitive response and in the acquired systemic resistance. Under normal watering conditions, SA was undetectable in mock-inoculated plants, while a remarkable boost in SA levels occurred in infected plants (Figure 6b). This accumulation pattern was opposite to that observed for ABA (Figure 6a), in agreement with the antagonistic activity of these two hormones [33]. This also indicated that, in the absence of abiotic stress, the plant response to TYLCSV infection is primarily mediated by SA rather than ABA. During water stress, the amount of SA increased in both groups of uninfected and infected plants, particularly in healthy individuals, but after recovery, SA became undetectable in both groups (Figure 6b). It is conceivable that the high SA accumulation measured in infected plants in normal watering conditions could exert a priming role, improving their ability to endure water stress effects. Moreover, the lower accumulation of SA during stress in TYLCSV-infected plants could be coherent with the results reported by [34], suggesting that an ABA increase associated to water deprivation is accompanied by a decrease in SA levels.

Due to the morphological alterations induced by TYLCSV on plant growth, including shoot, leaves, and root development, and considering the different xylem patterning above reported, changes in the amount of auxin were also evaluated. Our results attested that the pattern of IAA accumulation resembled that of ABA, showing a statistically significant increase upon water stress in both healthy and infected plants, followed by a sharp decrease during the recovery phase in both groups of plants (Figure 6c). However, regardless of the treatment, no statistically significant differences between uninfected and TYLCSV-infected plants were detected.

The similar concentration profile obtained for ABA and IAA is in agreement with the synergistic activity of these two hormones in the regulation of drought-induced tomato acclimation processes [35].

### 2.4. Virus Infection Induces Transcriptional Changes of Genes Regulating the Accumulation of Hormones and Drought-Related Metabolites

**Figure 7:**
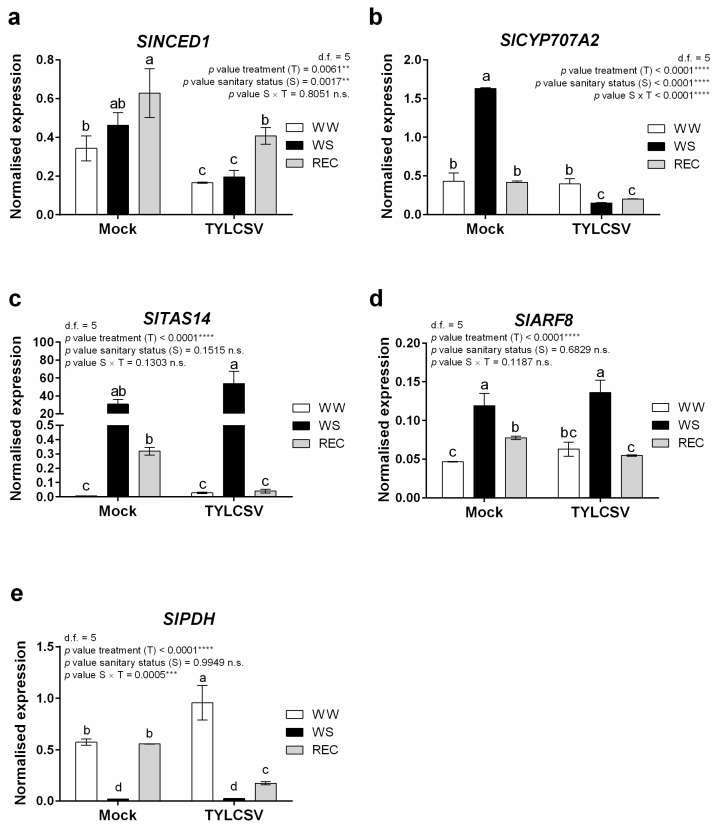
Expression changes of hormone-related and stress-responsive genes. Results of candidate gene expression analysis performed by RT-qPCR assay. Expression profiles of key genes involved in (**a**) ABA biosynthesis (*SlNCED1*) and (**b**) ABA degradation (*SlCYP707A2*), of genes encoding (**c**) the ABA-responsive dehydrin *SlTAS14*, and (**d**) the auxin response factor (ARF) *SlARF8*, and of genes involved in (**e**) proline degradation (*SlPDH*) in leaf samples of mock-inoculated or TYLCSV-infected plants, under irrigation (WW), water stress treatment (WS) or recovery (REC). Ubiquitin (*SlUBI*) and Elongation factor 1α (*SlEF*) genes were both used as endogenous housekeeping controls for the normalization of transcript levels. The significance of treatment (T), sanitary status (S), and sanitary status × treatment (S × T) interaction was assessed by Tukey’s *HSD* test and the corresponding results are given above each graph in the figure panel; *p* ≤ 0.01 (**); *p* ≤ 0.001 (***); *p* ≤ 0.0001 (****); n.s. = not significant. Lower case letters are reported when the S × T interaction and/or sanitary status (S) main effects are statistically significant, as attested by Tukey’s *HSD* or Student’s *t* test, respectively. Error bars represent SE. Three independent biological replicates with three technical replicates each were used for the analysis.

To support the above-reported hormone accumulation results and to better elucidate the modulation of the tomato plant response to the combined effect of virus infection and water deficit, we next investigated the transcriptional modulation of key genes that regulate hormonal balance and accumulation of drought-related metabolites, such as proline (Figure 7).

ABA metabolism is mainly governed by *SlNCED1*, encoding a 9-cis-epoxycarotenoid dioxygenase 1 and by *SlCYP707A2*, encoding a hydroxylase involved in its catabolism [36]. A progressive increase in the amount of *SlNCED1* occurred starting from the normal watering conditions to water stress and recovery, in both mock-inoculated and infected plants. However, despite the significant boost in ABA biosynthesis noticed upon drought (Figure 7a), infected plants overall showed significantly lower *SlNCED1* transcript levels compared to uninfected individuals (Figure 7a). Such discrepancy between molecular and hormone content data could rely on different balances in root-to-shoot ABA translocation events in plants encountering the two sanitary conditions [37]. Noticeably, in both sanitary conditions, the highest *SlNCED1* transcript levels were observed after rewatering, possibly suggesting that ABA-related molecular events governing the recovery phase were still active. Conversely, the expression level of the hydroxylase *SlCYP707A2* gene significantly increased in drought-stressed uninfected plants and decreased when stress was eliminated (Figure 7b). In TYLCSV-infected plants, a decrease of *SlCYP707A2* transcript levels occurred during water stress and recovery conditions (Figure 7b), showing a transcriptional profile opposite to that of *SlNCED1;* nonetheless, this transcriptional regulation could contribute to maintain a high ABA level (Figure 6a), favoring the virus-infected plant adaption to drought stress.

In addition, the transcript level of the ABA-related dehydrin-encoding transcript *SlTAS14* was evaluated, this gene being a well-known marker of drought stress response [38]. In fact, *SlTAS14* regulates the early accumulation of ABA in leaves and contributes to increase solute amounts in the cells in response to osmotic stress [39]. In line with this, a strong transcriptional activation of this gene was recorded in plants undergoing drought stress; an effect which was more pronounced in the case of TYLCSV-infected plants (Figure 7c). Moreover, while in normally hydrated plants no significant differences in *SlTAS14* expression were observed, upon recovery, only virus-infected plants restored the transcript levels to pre-stress conditions (Figure 7c). This finding further supports the concept that TYLCSV infection abbreviates the timing of drought recovery (Figure 2).

Members of the large *ARF* (auxin response factor) gene family are positive regulators of IAA production and play pivotal roles in plant growth and development [40] and in plant stress response [41]. Within the *ARF* gene family, *SlARF8* is highly expressed in tomato leaves [42] and has been recently shown to be transcriptionally regulated by drought and salinity [43]. In accordance with the results of IAA accumulation (Figure 6c), *SlARF8* transcription was significantly induced upon drought stress in both healthy and infected plants, and it was then restored to the pre-stress levels when plants recovered (Figure 7d).

To circumvent drought stress, plants also activate the production and accumulation of osmoprotectant compounds, including amino acids, proteins, sugars, and osmolytes such as proline [44]. In this context, we recently reported that healthy tomato plants undergoing a severe water stress treatment accumulate higher levels of the proline biosynthetic gene delta 1-pyrroline-5-carboxylate synthetase *SlP5CS1*, leading to increased production of proline in the leaf tissue [20]. Accordingly, to evaluate the impact of TYLCSV infection on tomato drought stress response, we analyzed here the transcriptional level of the proline degradation enzyme, the proline dehydrogenase *SlPDH* [45]. In well-watered conditions, *SlPDH* was significantly overexpressed in TYLCSV-infected plants compared to the uninfected group, while water stress induced a strong decrease in the gene expression in both sanitary conditions (Figure 7e). These expression changes are similar to those we previously reported for healthy non-transgenic plants irrigated and subjected to drought [20]. Interestingly, when recovery was completed, *SlPDH* transcripts amounts were significantly lower in TYLCSV-infected plants (Figure 7e), most likely underpinning reduced proline degradation levels during infection, therefore promoting reduced water stress perception of the plant. These results are partially in agreement with those reported by [16] showing that proline concentration was significantly lower in TYLCV-infected tomato plants in both well-watered and drought stressed conditions compared to healthy controls.

## 3. Materials and Methods

### 3.1. Biological Material Preparation

*Solanum lycopersicum* L. cv. Moneymaker plants (*n =* 12) were inoculated at the 4-leaf stage by injecting into the leaf axils aliquots of 30 µL of a suspension (OD600 > 10) of *Agrobacterium tumefaciens* strain LBA4404 carrying the agroinfectious 1.8mer TYLCSV construct (GenBank Acc. No. X61153), made in the pBin19 plasmid, as described in [46]. Another group of plants (*n = 12*) received agrobacteria containing the empty pBIN19 plasmid, hence serving as mock-inoculated controls. Plants were then scored for symptom development and, 2 months after inoculation (mpi), i.e., before the beginning of the drought stress imposition (see below), the presence of TYLCSV DNA was assessed by Southern blot hybridization, according to [47,48], using the Dot-Blot DNA extraction procedure outlined in [48].

### 3.2. Plant Growth and Experimental Design

Plants were grown in a glasshouse at an average daily temperature of 24.9 ± 5.35 °C and relative humidity levels ranging from 42.3 to 61.8%. Maximum photosynthetic photon flux density (PPFD) ranged between 900 and 1200 mol photons m^−2^ s^−1^ and illumination of 12-h-light/12-h dark-cycles was achieved using halogen lamps to provide a minimum PPFD of 500–600 mol photons m^−2^ s^−1^ during the light cycle. Each plant was kept in a 1.5-L pot filled with a sandy-loam soil/expanded clay/peat mixture (3:2:4 by volume). The same amount of soil (corresponding to 1 Kg) was loaded in each pot at plant transplantation. The drought experiment started at 2 mpi, using 12 mock-inoculated plants and 12 TYLCSV-infected individuals. Every morning for the whole duration of the experimental trial, half of the plants (*n* = 6) from each sanitary status were irrigated until pot water holding capacity (corresponding to 1 L volume), thus serving as WW controls, while another group of plants was subjected to complete water withdrawal (water stressed, WS; *n* = 6). Plants were monitored daily until the onset of severe WS symptoms (e.g., canopy collapse); at that time, WS plants were re-watered, using the same amount of water to both the TYLCSV-infected and mock-inoculated groups, and allowed to fully recover. A second independent drought and recovery time-course trial was performed in the same season, following the same experimental plan described above and using the same number of plants.

### 3.3. Morphological and Structural Analysis

Plant height, fresh shoot weight, and root length were measured in both healthy and infected plants at the beginning and at the end of the experiment, after recovery completion. Analysis of xylem transectional area was carried out as previously reported [20]. Briefly, freehand stem sections collected between the 7th and 8th leaf from the plant apex were stained for 2 min with an aqueous solution of 50 mg ml^−1^ safranin, washed 3 times with water and observed under a Leica DM 750 microscope equipped with an EC4 camera. The whole xylem area of the cross section was determined by elaborating microscope images with the ImageJ software (ImageJ, version 1.46r, National Institutes of Health, Bethesda, MD, USA, https://imagej.nih.gov). Three sections for each of four plants per condition (*n* = 12) were used for the analysis.

### 3.4. Measurement of Stem Water Potential

Stem water potential (*Ψ_stem_*) was measured for each plant on equilibrated non-transpiring (bagged) leaves. In brief, mature leaves were firstly covered with an aluminum foil and placed in a humidified plastic bag for at least 30 min before excision. After excision, leaves were allowed to equilibrate for more than 20 min in the dark; then leaves were analyzed for water potential using a Scholander-type pressure chamber (1505D PMS Instrument Company, Albany, OR, USA).

### 3.5. Hormone Content Analysis

The hormone content was measured as previously described [49]. Leaf samples (40 mg) were freeze-dried, homogenized, transferred to a 2-mL centrifuge tube, and extracted in an ultrasonic bath for 1 h with 1 mL of a methanol:water (1:1 *v*/*v*) mixture, acidified with 0.1% formic acid. After centrifuging the samples for 10 min at 15,000 rpm and 4 °C, the supernatant was used to quantify ABA, IAA, and SA, adopting the external standard technique, with calibration curves obtained using ABA (Sigma Aldrich, St Louis, MO, USA; purity 98.5%), IAA (Sigma Aldrich; purity ≥ 99%), and SA (Sigma Aldrich, purity ≥ 99%) original analytical standards. The HPLC-DAD equipment was an Agilent 1220 Infinity LC system model G4290B (Agilent^®^, Waldbronn, Germany), which included a gradient pump, autosampler, and a 30 °C column oven. A 170 Diode Array Detector (Gilson, Middleton, WI, USA) was used with a Nucleodur C18 analytical column (250 × 4.6 mm i.d., 5 μm, Macherey Nagel) set at 265 nm for both ABA and IAA and at 280 nm for SA. The mobile phases were water acidified with 0.1% formic acid (A) and acetonitrile (B), at a flow rate of 0.600 mL min^−1^ in gradient mode, 0–6 min: 30% of B, 6–16 min: from 30% to 100% B, 16–21 min: 100% B. Twenty μL per sample were injected, running three biological replicates for each condition; data are expressed as µg g^−1^ of dry weight.

### 3.6. Real Time PCR Analysis

Total RNA was extracted from 100 mg of powdered leaf tissue using the Trizol^®^ reagent (Invitrogen, Thermo Fisher Scientific, Waltham, MA, USA), according to the manufacturer’s instructions. RNA samples were then treated with TURBO DNase (Invitrogen, Thermo Fisher Scientific, Waltham, MA, USA) and the absence of genomic DNA was determined using the Qiagen One Step RT-PCR kit and 18S rRNA-specific primers. cDNA was synthetized from 1 μg of total RNA using the High-capacity cDNA reverse transcription kit (Applied Biosystems, Thermo Fisher Scientific, Waltham, MA, USA), according to the manufacturer’s procedure.

A CFX Connect Real-Time PCR system (Bio-Rad Laboratories, Hercules, CA, USA) was used to perform qRT-PCR (Bio-Rad), using 1 μL of 1:5 diluted cDNA, iTaq Universal SYBR Green Supermix (Bio-Rad), and 0.25 μM of each primer were used in the qPCR. Thermal cycling conditions were as follows: an initial denaturation at 95 °C for 10 min followed by 40 cycles at 95 °C for 15 s and 60 °C for 1 min. Primer specificity was inspected by running a dissociation kinetics curve at the end of each qPCR run. The 2^−ΔCt^ method [50] was used to calculate the expression levels of tomato target transcripts, following normalization to the geometric mean of the Elongation factor (*SlEF*) and Ubiquitin (*SlUBI*) transcripts used as endogenous controls. Each sample was examined in three biological replicates using three technical replicates for each run. Gene-specific primers used in real-time qPCR assays are as described in [20].

### 3.7. Statistical Analyses

Significant differences among treatments and plant sanitary condition were determined by running a two-way analysis of variance (ANOVA). When results of the ANOVA test pointed that either sanitary status (S: ‘M’, ‘TYLCSV’) or treatment (T: WW, WS, REC) or their interaction (S × T) was significant, the Tukey’s honestly significant difference (*HSD*) post-hoc test was used to separate the means (*p* < 0.05). The S main effects were statistically determined by a two-tailed Student’s *t* test. The SPSS statistical software package (SPSS Inc., Cary, NC, USA, v.22) and the GraphPad Prism software (GraphPad Software, La Jolla, CA, USA, v.6.01) were used to perform the statical elaborations and plot figure charts, respectively.

## 4. Conclusions

Understanding the mechanisms that support water stress tolerance in tomato plants is fundamental due to the huge economic and commercial importance of this vegetable crop, considering in particular the future projected environmental change scenarios [51]. In this work, we show that tomato infection by TYLCSV, a representative species inducing one of the most devastating diseases affecting this crop in the Mediterranean area, contributes to promote water stress tolerance. The findings that TYLCSV-infected plants perceive the stress later and recover faster after a period of complete water deprivation compared to healthy individuals are supported not only by anatomical observations of the xylem vasculature (i.e., reduced xylem cross-sectional area) but also by changes in the expression of key ABA- and drought-stress responsive genes. Moreover, measurements of ABA and SA content further point to the induction of a primed condition of infected plants towards stress management. The present study confirms observations made by other groups regarding the increased abiotic stress tolerance of plants infected by viral agents [4,8,10], including the reports by [15,16,17] describing the drought stress mitigation in plants infected by another TYLCD-inducing virus. Different mechanisms can concur to activate such host resilience empowerment, including increased production of antioxidant enzymes, unbalance of phytohormone levels, increased water and nutrient uptake as well as upregulation of stress-responsive genes [10]. Here, alterations in hormone levels, particularly ABA, and anatomical modifications connected to water stress management have been described, but further elucidation of the interaction between viruses and plants exposed to drought stress, and precise identification of the ongoing biochemical and molecular events are awaiting.

Due to the heavy impact of TYLCSV on tomato crop yield, such virus-mediated beneficial effects are hard to be practically exploited. Nonetheless, several tomato cultivars or hybrids carrying genes that confer resistance to viruses inducing TYLCD are currently commercialized and cultivated to reduce yield losses [52,53]. Importantly, these lines are not immune to viral infection but typically develop systemic infection with mild symptomatology, sufficient to allow commercialization of tomato fruits [52]. Furthermore, since up to 13 viral species are reported to induce TYLCD, also in association with satellites [54], it would be interesting to verify if these species also contribute to increase the mitigation of stress response in the tomato host.

As already proposed by H. Czosnek and co-workers [17] in the tomato-TYLCV context, the pathophysiological behavior of TYLCSV-tolerant tomato lines in conditions of water deprivation represents the next step to investigate, in order to move towards the exploitation of the achievements reported here. Additionally, expanding this concept to other pathosystems, i.e., evaluating if other geminiviruses enhance resilience of their hosts to abiotic stresses would not only deepen the global knowledge of the cross-talk between biotic and abiotic stress perception and response, but it will also represent a key step towards future genetic agronomical applications.

The exploitation of viruses to bolster plant drought resilience can also have relevant evolutionary implications. Indeed, co-evolution events of a plant virus hosted by drought-stressed *Arabidopsis* plants led to the development of variants, providing plants with additional drought tolerance [55]. In this scenario, the recombination events frequently occurring within geminivirus populations should be explored in terms of possible increased beneficial effects conferred to their hosts.

We recently proposed that transgenic overexpression of the TYLCSV C4 protein is a major driver of the ameliorated performances of the tomato towards drought stress [20]. Further transcriptomic and metabolomic studies are expected to provide a clearer picture of the biology underpinning the role of virus infection on the plant’s ability to withstand abiotic stresses.

Overall, this study further contributes to widen the general understanding of the virus—host cross-talk and its resulting impact on the plant’s adaptability to environmental stress. On a broader perspective, our outcomes are particularly relevant in the current scenario of climate change and support deeper investigations addressed to develop novel agronomic practices of cultivating crops in locations with limited access to water and with high temperatures.

## Data Availability

All the data are available in the manuscript.
